# Clinical Efficacy of Two Different Desensitizers in Reducing Postoperative Sensitivity Following Composite Restorations

**DOI:** 10.7759/cureus.25977

**Published:** 2022-06-15

**Authors:** Rutuja Rajnekar, Nikhil Mankar, Pradnya Nikhade, Manoj Chandak, Anuja Ikhar, Karuna Burde

**Affiliations:** 1 Conservative Dentistry and Endodontics, Sharad Pawar Dental College, Datta Meghe Institute of Medical Sciences (DMIMS) Deemed University, Wardha, IND; 2 Public Health Dentistry, Saraswati Dhanvantari Dental College and Hospital, Parbhani, IND

**Keywords:** class i restoration, desensitizing agents, gluma, composite, postoperative sensitivity

## Abstract

Objective: The objective is to evaluate the efficacy of different desensitizing agents in the reduction of postoperative sensitivity after composite restoration.

Materials and methods: Class I cavities were prepared in 39 patients by the same operator. The patients included in the study were between 20 and 45 years with vital pulp and a remaining dentin thickness of 1mm. Previously restored, nonvital and tooth with periodical changes were excluded. Patients were randomly assigned into three groups of 13 each - Group I (Control); Group II (Gluma desensitizer) and Group III (shield active desensitizer [SAD]). After the surface treatment, the teeth were restored with composite. The patients were assessed for postoperative sensitivity at 24 hours and one week with a visual analog scale (VAS).

Statistical Analysis: Data were analyzed using SPSS v23 software. Data were analyzed using one-way ANOVA and post-hoc Tukey test.

Results: Gluma and SAD on comparison with control group i.e. composite group showed statistical significance difference at day 1 (P-value 0.003), but on comparing the sensitivity after one week, there is no significant difference in sensitivity score between all three groups (P-value 0.073). There was no statistically significant difference between day 1 and one week when comparing Gluma desensitizer and SAD.

Conclusion: The application of the desensitizer led to a statistically significant reduction in postoperative sensitivity on day 1 and a clinically significant reduction was observed at one week.

## Introduction

The field of dentistry has made significant advances in the past few decades, including advances in restorative dentistry where newer materials have been developed that form a mechanical bond with the tooth and require conservative cavity preparation [1]. With the newer adhesive materials, enamel and dentin bond effectively. Despite the availability of newer materials and improvements in composite properties, postoperative sensitivity remains a major concern for a dentist after composite restoration placement. It has been observed that patients complain of dentinal sensitivity at different levels and in different situations, especially in posterior teeth. Even without visible failures in the restoration, it is a common problem. The term post-operative sensitivity refers to “pain in a tooth associated with mastication or with sensitivity to hot, cold, and sweet stimuli that occur one week or more after restoration” [2]. According to several clinical studies, nearly 30% of patients report postoperative sensitivity after placing resin composite restorations in posterior teeth [3]. It has been widely accepted that tooth sensitivity is caused by the “hydrodynamic theory,” developed in the 1960s on the basis of years of research. In accordance with the hydrodynamic theory “when the fluid within the dentinal tubules is subjected to changes in temperature or osmotic pressure, the movement stimulates the nerve receptor sensitive to pressure, which results in the transmission of the stimuli” [4]. Despite the success of the enamel bonding technique introduced by Buonocore in 1955, problems have been experienced with dentine bonding due to its moist organic composition [5].

Postoperative sensitivity can be explained by several factors. When the dentinal tubules are open in numerous numbers, the adverse effects because of cavity preparation, like dentine dehydration and excessive heat, are more likely to reach the pulp [6]. The condition is exacerbated when the dentin is acid-etched. When acid etching is performed, not only does it widen tubules, but it also removes the smear layer, thereby physically sealing them off from external stimuli.

The sensitivity usually persists for a period of a few days to several months before subsiding completely. Rarely in a few cases, there may be pulp involvement which may lead to endodontic treatment. Despite dealing with occlusal interferences, most sensitivity is present at the edges of restoration and sometimes at the center. In terms of technique, instrument, and material, Composite resin-based fillings are sensitive restorations since they bond to the tooth structure mechanically and/or chemically. As composite resins can cause pulp irritation, they should be bonded carefully, especially in deep dentinal cavities along with further use of a desensitizing agent, liner, and resin-modified glass ionomer where necessary, for preventing postoperative sensitivity and subsequent pulpal death; as total-etch bonding system could cause a detrimental effect on the pulp [7].

Dental desensitizers are often used to treat postoperative sensitivity. By obliterating the dentinal tubules, the dentin desensitizers reduce hypersensitivity [8]. Many methods are employed to desensitize the dentin, including anti-inflammatory medications, adhesives, varnishes, tubular obliterating procedures, and lasers.

Gluma desensitizer (Heraeus Kulzer) is a glutaraldehyde-based hydroxyethyl methacrylate (HEMA) containing a substance that blocks dentinal tubules by coagulating the plasma proteins within them [9]. SAD (Prevest Denpro Ltd, India), contains 2-hydroxyethyl-methacrylate, benzalkonium chloride, sodium fluoride, potassium nitrate, and excipients. The component HEMA penetrates the dentinal tubules and physically seals them. This study aimed to determine the effectiveness of Gluma desensitizer and SAD in preventing postoperative sensitivity in Class I composite restorations.

## Materials and methods

Study design and enrollment

The ethical approval was obtained to perform the study from Datta Meghe Institute Medical Ethical Committee (DMIMS(DU)/IEC/2021/420). Class I cavities from 39 patients were included in the study from August 2021 to December 2021. After explaining the procedure, consent was taken for the study. The inclusion criteria were - the study included a group of patients aged 20-45 with vital pulp and a remaining dentin thickness of at least 1 mm. The exclusion criteria were previously restored, nonvital, and tooth with periodical changes.

Sample size determination

Depending on the mean and standard deviation of previous studies, a sample size of 13 was calculated for each group.

Allocation

By using computer-generated simple random sampling, the desensitizers were assigned to the patients. The numbers were randomly selected, with each number representing either a desensitizer or the control group. The two desensitizers which were used in the current study were Gluma desensitizer and SAD. The three groups, comprising 13 patients each, were as follows: Group I: No desensitizer was applied before the restoration (control group), (n = 13); Group II: Gluma desensitizer was applied before the restoration (n = 13); Group III: SAD was applied before the restoration (n = 13).

Blinding

After completion of informed consent, a number was assigned to each participant by the assessor (NM) who was blinded to the procedure to be performed. A blinded approach was used for assigning patients to the control or one of the desensitizer groups. Desensitizers (or control) were applied by a single, well-trained operator (RR). As a result, the randomization process and the application of the desensitizing agents were conducted by the same operator. During the study, the sensitivity scores were assessed and recorded by patients blind to the random allotment process and protocol, making the study double-blinded.

Clinical procedures

Following anesthesia, for the isolation of the tooth, the rubber dam application was done. Class I cavity were prepared using a round BR 45 and NO.245 straight fissure diamond bur (MANI Inc., Japan) in a high‑speed air‑turbine handpiece (NSK INC, Japan) with copious water irrigation. After cavity preparation, for acid etching 37% phosphoric acid (Total‑etch, Ivoclar Vivadent) was applied to cavosurface enamel for 15 sec, and after that etchant was applied to dentin for 10 sec. The cavity was washed thoroughly for 1 min with water. The entire cavity was gently moist‑dried to remove the surface moisture. For Group I (Control group) the entire cavity was coated with a Bonding agent (Tetric N bond, Ivoclar Vivadent), the excess was removed and light-cured for 15 sec. The cavity was restored with a nanohybrid composite (Tetric N Ceram, Ivoclar Vivadent). The occlusion was checked. During the same visit, the resin surfaces were accessed, finished, and polished. In both the desensitizer groups, after acid etching, the desensitizers were applied with a disposable brush and were maintained over the dentine for 1 min. After removing the excess with a gentle stream of air, the cavity was cleaned and dried. Following this similar restorative protocol was followed as in the control group.

Assessment

Afterward, all the participants were briefed regarding the Visual analog scale (i.e., VAS) and trained by a single instructor to mark the pain intensity experienced by them after 24 hrs and one week. Patients were provided with telephone assistance for recording postoperative pain on the VAS at different intervals. VAS had a score of 0 to 10, 0 and 1 ‑ no sensitivity; 2 and 3 ‑ mild, annoying sensitivity; 4 and 5 ‑ nagging, uncomfortable and troublesome sensitivity; 6 and 7 ‑ distressing and miserable sensitivity; 8 and 9 ‑ intense, dreadful and horrible sensitivity; 10 ‑ worst possible, unbearable sensitivity.

Statistical analysis

Data were analyzed using SPSS v23 software. The level of significance was kept at 5%. Changes in dentinal hypersensitivity from day 1 to day 7 within each group were analyzed using paired t-test. Intergroup comparison of dentinal hypersensitivity was performed using one-way ANOVA test and post-hoc Tukey test.

## Results

**Figure 1:**
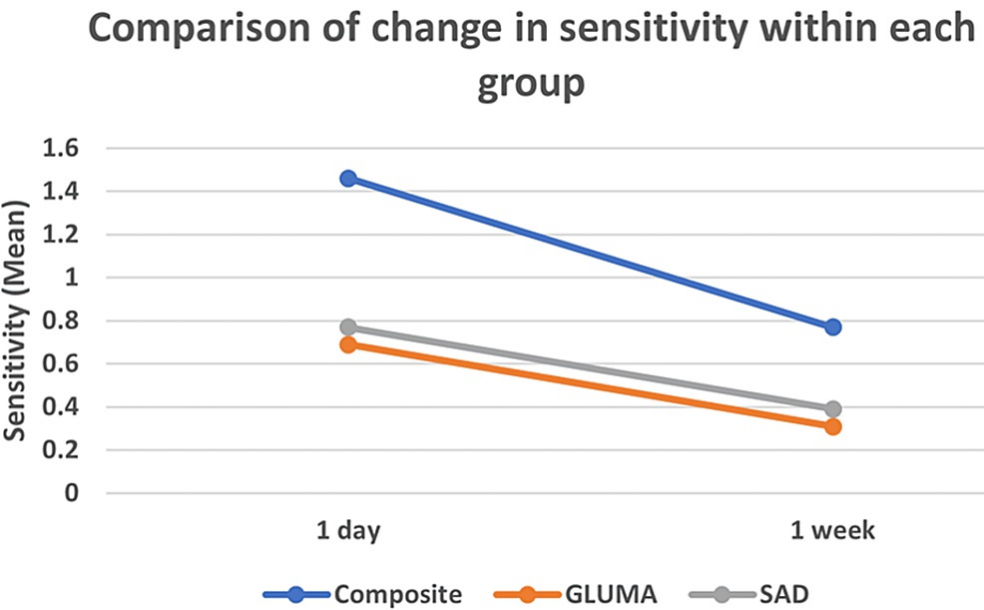
Comparison of change in sensitivity within each group Comparison of change in sensitivity within each group

There was a statistically significant difference for each group (i.e., composite; Gluma; SAD) for change in sensitivity between 24 hrs and one week (Table 1, Figure 1). It was observed that Gluma showed better results (P-value 0.049) in reducing postoperative sensitivity compared to the SAD and composite group.

**Table 1:** Comparison of change in sensitivity within each group Comparison of change in sensitivity within each group Paired t-test; * indicates a significant difference at p≤0.05

Groups	1 day		1 week		Difference	P-value
	Mean	SD	Mean	SD		
Composite	1.46	0.78	0.77	0.60	0.69	0.032*
GLUMA	0.69	0.48	0.31	0.48	0.38	0.049*
SAD	0.77	0.44	0.39	0.51	0.38	0.018*

**Figure 2:**
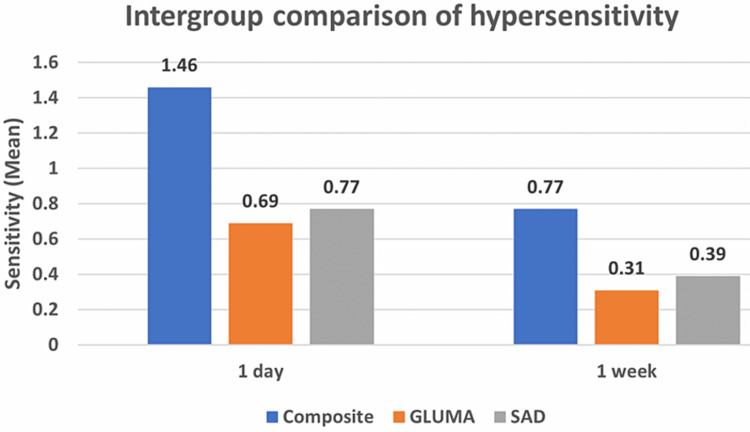
Comparison of sensitivity score between three groups Comparison of sensitivity score between three groups

In current study on comparison of sensitivity score between all three groups, it showed statistical significance difference at day 1 (P-value 0.003), but on comparing the sensitivity after one week the there is no significant difference in sensitivity score in all three groups (P-value 0.073) using one-way ANOVA test (Table 2, Figure 2). The results showed that the postoperative composite sensitivity decreases with time.

**Table 2:** Comparison of sensitivity score between three groups Comparison of sensitivity scores between three groups One-way ANOVA test; * indicates a significant difference at p≤0.05 SAD - shield active desensitizer

Groups	Composite		Gluma		SAD		P-value
	Mean	SD	Mean	SD	Mean	SD	
1 day	1.46	0.78	0.69	0.48	0.77	0.44	0.003*
1 week	0.77	0.60	0.31	0.48	0.39	0.51	0.073 (NS)

When Intergroup comparison was done it showed statistically significant difference at day 1 between Composite and Gluma (P-value 0.005); Composite and SAD (P-value 0.013). However, no statistically significant difference was observed between Gluma and SAD (P-value 0.940) at day 1. On comparison for one week, no statistically significant difference was observed between composite and Gluma (P-value 0.082); Composite and SAD (P-value 0.169); Gluma and SAD (P-value 0.928). But reduction in sensitivity for Gluma was better than SAD (Table 3).

**Table 3:** Pairwise comparison of sensitivity score between three groups Post-hoc Tukey test; * indicates significant difference at p≤0.05 SAD - shield active desensitizer

Groups	Composite vs GLUMA		Composite vs SAD		GLUMA vs SAD	
	Difference	p value	Difference	p value	Difference	p value
1 day	0.77	0.005*	0.69	0.013*	-0.08	0.940 (NS)
1 week	0.46	0.082 (NS)	0.38	0.169 (NS)	-0.08	0.928 (NS)

## Discussion

The advent of newer techniques and concepts in adhesive dentistry has increased the number of composite restorations done. Several studies have identified the three most common causes of postoperative sensitivity related to composite restoration: polymerization shrinkage of the resin, microleakage around the margins of the restoration, and residual stress in the tooth structure after the placement of direct composite restoration [10]. More than 6% of complaints of sensitivity to cold and hot drinks and mastication after direct composite resin restorations can be attributed to increased cavity depth [11].

Etching dentin and the formation of a hybrid layer introduced by Nakabyashi in 1992 were promising theoretically, but the related complications of etching created quite a few complications practically, one of which being the post‑operative sensitivity. Hence, the self‑etch concept came into practice but still never gave a complete solution.

Another issue with bonded interface during polymerization is excessive stress that leads to dentin crack formation on the pulpal floor and sensitivity while chewing. Despite modifications to surface treating the tooth, incremental placement of composite, and changes in polymerization techniques, the problem of post‑op sensitivity still remains.

Studies evaluating the prevention of postoperative sensitivity after composite restoration are few. Dentinal desensitizers are used to reduce the sensitivity resulting from composite restorations. When applied in the cavity before dental adhesives, they do not affect the bonding of the restorative material. This allows composites to adhere to cavity walls without interfering with their adhesion and sealing.

HEMA, a component of Gluma desensitizer and SAD, may serve as a carrier/wetting agent for glutaraldehyde and benzalkonium chloride. HEMA penetrates the dentinal tubules and physically seals them. As HEMA is soluble in water, it allows glutaraldehyde to penetrate the tubules and allows for in-depth action. Furthermore, glutaraldehyde inhibits the growth or invasion of bacteria through the interface between the tooth and the restoration when there is a sealing imperfection. Additionally, glutaraldehyde also functions as a biological fixative by coagulating the plasmatic proteins of the dentinal fluid inside the tubule [12]; it forms septa which prevent the shifting of pulp fluid inside the tubule. As a result of this complex mechanism, the dentine should be impermeable by canceling the hydrodynamic effect and, therefore, desensitize the teeth. Also, the benzalkonium chloride serves as an antimicrobial agent and it participates in the cross-linking process associated with dentinal bonding.

In the current study, we have compared effect of two desensitizers, i.e., Gluma and SAD, in an in vivo clinical trial at one day and after one week. The VAS was used to evaluate the subjective perception of post-operative sensitivity. 0-10 numerical scores were considered, which is the usual method of scoring pain. It was observed that with time, sensitivity decreases. At day 1, a significant reduction in pain was observed in all groups except the control group with desensitizers (p≤0.05). However after one week, no statistically significant difference was observed between sensitivity scores, indicating sensitivity decreases with time. But overall Gluma performed better than the control and SAD group. This was in accordance with previous studies conducted by Dondi and Malferrari; Patil et al. and Sayed et al. confirmed that Gluma desensitizer significantly lead to reduction of postoperative sensitivity [13,14]. Despite these results, no significant difference was observed in the clinical study conducted by Sobral et al. with Gluma for any of the stimulus [15]. The study proposed that the desensitizer application reduced the post-restorative sensitivity in the composite restorations.

According to this study, only a small percentage of restored teeth undergo postoperative sensitivity when the restorative procedure is properly performed. In the investigation, every step in the restoration process were carefully evaluated, from radiographic examination, pulp testing, polishing. There were no statistically significant differences between the three treatments given in this study, which may explain the results reported. According to Opdam et al., postoperative sensitivity, one of the major factors determining a composite resin restoration's clinical success, has been shown to be significantly influenced by the restorative technique employed by the clinician [16].

In this study, it was found that there was significant reduction in post‑op sensitivity in the patients where desensitizing agent was applied before adhesive protocols. The total-etch technique can be improved with tooth-desensitizing solutions. These solutions should be used following the completion of acid etching and after the tooth surfaces have been dried slightly. In the desensitizing solutions, the hydroxyl ethyl methacrylate wetting the tooth surfaces, followed by the subsequent drawing of the bonding resin into the dentinal canals, results in a reduction in tooth sensitivity significantly [17]. And this could be the reason for the reduction of post‑op sensitivity.

Limitations of the present study include its short duration and limited sample size. The study was performed in Class I cavity design and followed up for one week. Studies with glutaraldehyde and HEMA have demonstrated cytotoxic effects, raising questions about the biocompatibility of the desensitizer agent components [18,19]. Further research is needed to determine the cytotoxic potential of desensitizers. Moreover, long-term, multi-center clinical trials should be conducted in the future to confirm the present findings.

## Conclusions

Management of postoperative sensitivity is essential for the success of dental composite restorations. Under the limitations of the study, it was found that there was a significant reduction in post‑op sensitivity in patients who underwent surface treatment before adhesive protocols. In order to better understand the efficacy of desensitizers, a longer follow-up with different designs is recommended,
